# Convenient Preparation of PEDOT-Based Conductive Fabrics via a Green Strategy for Morse Code Recognition

**DOI:** 10.3390/polym17131816

**Published:** 2025-06-29

**Authors:** Hongjian Yu, Yifan Cui, Miao Miao

**Affiliations:** 1 College of Light Industry and Textile, Qiqihar University, Qiqihar 161006, China; qqhuyhj@163.com; 2 Xinjiang Production & Construction Corps Key Laboratory of Modern Textile Materials and Technology, College of Fashion and Textile, Tarim University, Alaer 843300, China; 3 State Key Laboratory of Separation Membranes and Membrane Processes, School of Textile Science and Engineering, Tiangong University, Tianjin 300387, China

**Keywords:** doping amount, enzymatic polymerization, horseradish peroxidase, conductive polymer, pressure sensor, morse code

## Abstract

With the rapid development of Internet of Things (IoT) and bioscience technology, wearable smart devices are developing toward advanced trends such as flexibility, convenience and environmental-friendliness. Poly (p-styrenesulfonic acid) (PSS), as a common template and dispersant, is indispensable in the polymerization of conductive polymers. However, the doping amount of PSS has a significant effect on the electrical conductivity of the polymer. Herein, different molar quantities of PSS were used to assist the polymerization of 3,4-ethylenedioxythiophene (EDOT) monomer in a horseradish peroxidase/hydrogen peroxide (HRP/H_2_O_2_) low-temperature system to obtain conductive finishing solutions with more excellent electrical properties. Then, the polyester nonwoven fabric was immersed in the conductive finishing solution, and when the addition ratio of EDOT and PSS was 1:2, the conductive performance was optimal (3.27 KΩ cm^−1^). Finally, the conductive fabric was assembled into a pressure sensor and a temperature sensor, which can transmit Morse code in the form of single-parameter (pressure response or temperature response) or collaboration. Overall, this research has great potential for production of poly(3,4-ethylenedioxythiophene) (PEDOT)-based composites and their applications in smart wearable device.

## 1. Introduction

Currently, the design and development of flexible electronic devices have attracted increasing attention by virtue of the advantages of textiles (comfortable to wear, breathable, and soft) [1,2]. Conductive polymers have been widely utilized as conductive layers in textile-based electronic devices due to their excellent mechanical properties and ease of processing. Generally, the preparation methods of conductive fabrics, which involve depositing conductive polymers onto textile surfaces, include dip-coating [3], vapor-phase deposition [4], electrochemical deposition [5], in-situ solution polymerization [6], and drop-injection [7]. Among these conductive polymers (such as polyaniline (PANI) [8,9], polypyrrole (PPy) [10,11], poly(3,4-ethylenedioxythiophene) (PEDOT) [12,13,14] and their derivatives [15,16]), PEDOT is considered as an ideal material for conductive layers due to its low cost, high conductivity, excellent environmental stability and promising commercial applications [17].

PEDOT can be synthesized by chemical, electrochemical and enzymatic polymerization. Enzymatic polymerization has the advantages of high efficiency, catalytic specificity, mild reaction conditions and environmental friendliness [12]. Natural enzymes such as horseradish peroxidase (HRP) [18], laccase [19] and soybean peroxidase (SBP) [20] can be used for the synthesis of PEDOT. Generally, templating agents are required for the synthesis of PEDOT. Generally, negatively charged linear polymers can serve as templates for the synthesis of PEDOT, such as poly(p-styrenesulfonic acid) (PSS) [21,22], sodium dodecyl sulfate (SDS) [23], sodium lignosulfonate (LS) [24], and p-toluenesulfonic acid (TsOH) [6]. Currently, numerous studies have focused on using water-soluble PSS as a template to assist in the polymerization of 3,4-ethylenedioxythiophene (EDOT) monomers. However, adding non-conductive PSS to PEDOT systems may affect the conductivity of the products. In previous research, a one-step enzymatic in-situ polymerization method was used to directly assemble PEDOT on the substrate surface with PSS as both the template and dopant [21]. This approach, however, yielded small sample sizes and required lengthy reaction times, hindering large-scale production. Therefore, investigating the impact of dopant content on the conductivity of poly(3,4-ethylenedioxythiophene):poly(styrene sulfonic acid) (PEDOT:PSS) represents a valuable research direction.

In this study, a green and eco-friendly HRP/H_2_O_2_ biocatalytic system was developed to synthesize PEDOT:PSS finishing solutions doped with varying molar amounts of the templating agent (PSS), with the aim of investigating the effect of doping levels on the conductivity of the resulting solutions. Additionally, pressure sensors and temperature sensors were prepared by coating the PEDOT:PSS finishing solutions onto nonwoven fabrics, and their capabilities for signal monitoring and transmission were evaluated. This research provides a new approach for mechanistic studies of conductive polymers and offers novel possibilities for the convenient fabrication of wearable electronic sensors.

## 2. Experimental Section

### 2.1. Materials and Reagents

PSS (Mw~75,000, 18 wt% in H_2_O) and EDOT (99%) were acquired from Sigma-Aldrich (St. Louis, MO, USA). HRP (RZ: >2.0, freeze-dried powder, ≥300 units/mg) were acquired from Aladdin. H_2_O_2_ (≥30.0 wt%) was obtained from Sinopharm Chemical Reagent Co., Ltd. In addition, all of the reagents were analytical grade, were directly used without further purification, and were prepared with deionized water. PET nonwoven fabrics were purchased from Foshan Jiada Nonwoven Fabrics Co., Ltd. (Foshan, China), and were pretreated with deionized water and anhydrous ethanol.

### 2.2. Biocatalytic Preparation of PEDOT:PSS Conductive Finishing Solution

**Figure 1:**
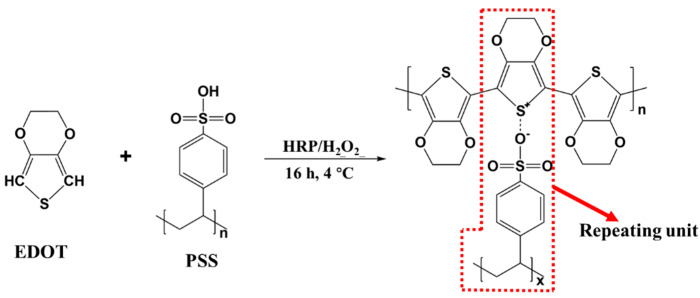
Scheme of enzymatic-catalyzed polymerization of PEDOT:PSS with HRP/H_2_O_2_ catalytic system.

The polymerization of EDOT was catalyzed by HRP and the oxidizing agent (H_2_O_2_) under 4 °C (Figure 1). In brief, EDOT and PSS were added in 20 mL deionized water in the constant low-temperature device. After the solution homogeneous mixing, 6 mg of HRP solution (3 mg/mL of deionized water) and 50 mM of H_2_O_2_ solution were added and mixed. The reaction was carried out for 16 h to complete the polymerization process, and a characteristic dark-blue of PEDOT:PSS conductive finishing solution was obtained. The addition proportion of PSS & EDOT and the products’ names were shown in Table 1.

### 2.3. Preparation of PEDOT:PSS Nonwoven Fabric and Assemble Devices

Firstly, commercialized nonwoven fabric was washed by alcohol and acetone to take away impurity substance. The washed nonwoven fabric was then dished in PEDOT:PSS conductive finishing solution and deposited for 1 h after 30 min ultrasonication. Finally, the modified nonwoven fabric was dried in an oven at 40 °C and designated as M-nonwoven-X, where X represents the molar ratio of PSS to EDOT. Among these samples, M-nonwoven-12 was selected for assembling the pressure sensor and the self-powered movement keyboard.

### 2.4. Characterization

UV–vis spectra were recorded on a spectrometer (UV-2808S, Shanghai Sunny Hengping Scientific Instrument Co., Ltd., Shanghai China) in the range of 200–1100 nm. In each measurement, deionized water was used as the control. Fourier transform infrared (FT-IR) (Nicolet iS 10, Thermo Fisher Scientific, Waltham, MA, USA) spectra were recorded on a spectrometer (at attenuated total reflection mode (ATR) with 32 scans and 4 cm^−1^ resolution in the scanning range of 4000–400 cm^−1^). Raman spectra were recorded on a spectrometer (inVia Reflex, Renishaw plc, Wotton-under-Edge, UK). Collecting 5 spectra on different points of the sample on glass coupled. The laser wavelength was 532 nm and a range from 100 to 3100 cm^−1^. Scanning electron microscopy (SEM) (JEOL Ltd., Akishima, Japan) was used to observe the microscopic view at 10 KV.

### 2.5. Electrical Property, Signal Monitoring, and Transmission Measurement

To examine the effect of the addition proportion of PSS and EDOT on electrical conductivity, electrical resistance of the modified nonwoven fabric was measured utilizing a digital precision multimeter (DMM 6550, Tektronix Inc., Solon, OH, USA). The signals were recorded in real-time using a Keithley 6550 multimeter. Each data point was measured twenty times, and the average value was subsequently recorded.

### 2.6. Pressure Sensor Assembly and Mechanism

**Figure 2:**
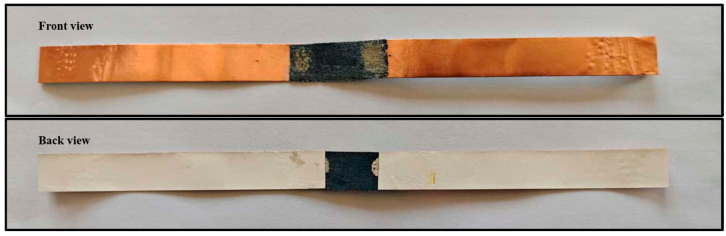
Physical images of pressure and temperature sensor devices.

The pressure sensor was fabricated by assembling a 1 cm × 3 cm strip of PEDOT-based conductive fabric using conductive silver paste and copper tape (Figure 2). When a finger adhered with a 2 cm × 2 cm patch of PEDOT-based conductive fabric presses the sensor, the instantaneous resistance decreases due to the creation of additional conductive paths. Specifically, the duration of each press directly translates into Morse code elements: a short press denotes a “dot” (·), while a long press signifies a “dash” (–).

### 2.7. Temperature Sensor Assembly and Mechanism

The temperature sensor was fabricated from a 1 cm × 3 cm strip of PEDOT-based conductive fabric, bonded with conductive silver paste and copper tape (Figure 2). The two ends of the strip serve as the hot and cold junctions, respectively. When the hot junction rapidly touches and separates from a heat source, it generates a positive voltage pulse corresponding to a “dot” (·) in Morse code. Conversely, maintaining prolonged contact produces a sustained positive voltage signal representing a “dash” (–). By modulating the thermal contact timing, various alphanumeric characters can be encoded through combinations of these “dot-dash” signals.

## 3. Results and Discussion

### 3.1. Chemical Structure of the PEDOT:PSS and Modified Nonwoven Fabric

**Figure 3:**
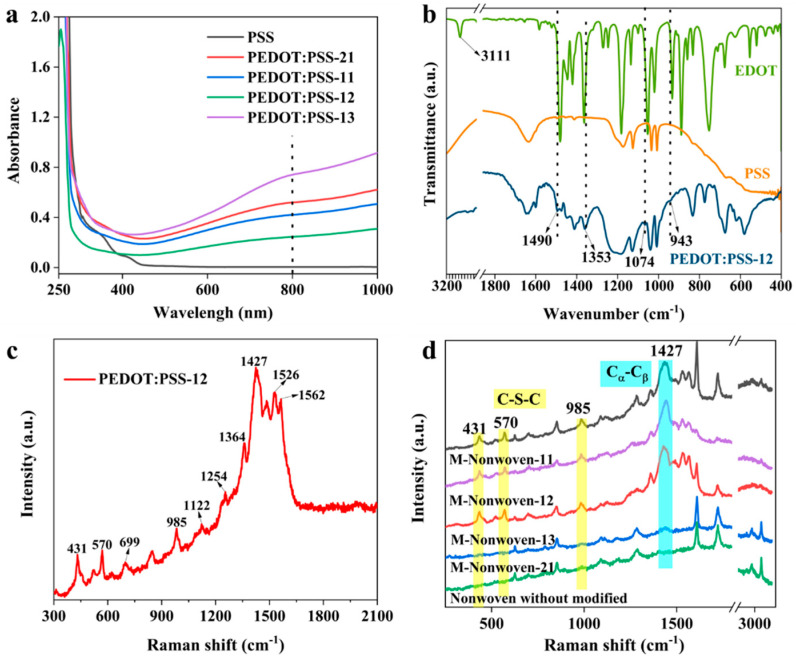
(**a**) The absorption spectra of PEDOT:PSS with different addition proportion. (**b**) The FT-IR spectra of EDOT, PSS and PEDOT:PSS-12. (**c**) The Raman spectrum of PEDOT:PSS-12. (**d**) The Raman spectra of nonwoven fabrics before and after modified.

The UV-Vis absorption spectra of obtained PEDOT:PSS with different addition proportions were shown in Figure 3a. As shown in the figure, the template agent PSS exhibits no significant absorption above 400 nm compared to PEDOT:PSS. The absorption bands of PEDOT:PSS in the range of 600–800 nm are attributed to π–π* transitions occurring within the PEDOT polymer chains [25]. To determine the chemical structure of the polymer, Figure 3b shows the FT-IR spectra of EDOT, PSS, and PEDOT:PSS-12. The characteristic peak (3111 cm^−1^) of the EDOT monomer appears in FT-IR spectra, it is due to the C-H band stretching in the thiophene ring. However, the most notable feature was the absence of a peak at 3111 cm^−1^ in the PEDOT:PSS spectrum, which indicates the successful enzymatic polymerization of PEDOT. Furthermore, the characteristic bands of PEDOT were also observed. The classical band C=C antisymmetric stretching, C=C symmetric stretching, and C-C stretching of the thiophene ring are between 1353 cm^−1^ and 1490 cm^−1^, C-O stretching at 1074 cm^−1^ and C-S stretching at 943 cm^−1^ [5,26].

To check the presence of PEDOT:PSS on nonwoven fabric, the samples were observed by Raman spectroscopy (Figure 3c,d). The Raman bands observed at 431, 570, and 985 cm^−1^ correspond to the deformation vibrations of the polyethylene ring, while those at 699 and 1122 cm^−1^ are attributed to the C–S–C and C–O–C bending modes, respectively. The bands at 1254 and 1364 cm^−1^ were C_α_-C_α_ inter-ring stretching and C_β_-C_β_ stretching, respectively. The bands at 1427, 1526, and 1562 cm^−1^ were the C_α_-C_β_ symmetric stretching and C_α_-C_β_ asymmetric mode, respectively. However, the spectrum of pristine PET was in absence of the C-S-C absorption band, the C_α_-C_β_ symmetric stretching, and C_α_-C_β_ asymmetric mode. This indicates that PEDOT:PSS was successfully deposited on nonwoven fabrics.

### 3.2. Surface Morphology and Composition of the PEDOT:PSS Modified Nonwoven Fabric

**Figure 4:**
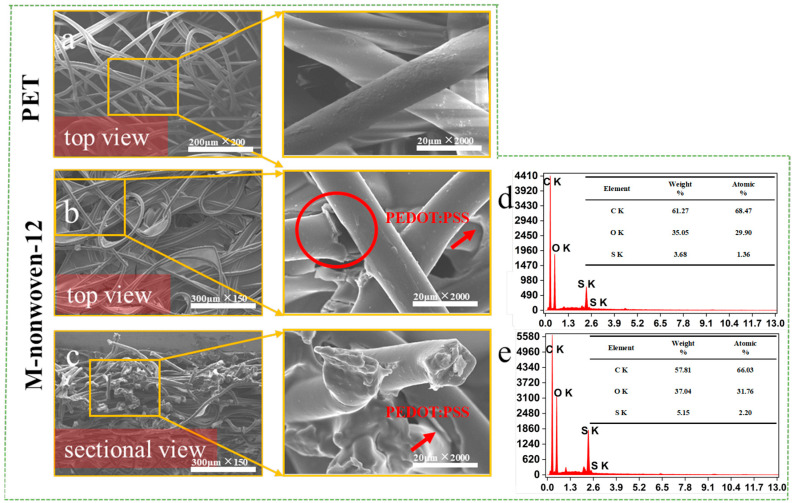
The SEM images of nonwoven fabric (**a**), and M-nonwoven-12 (**b**,**c**). The EDS image of M-nonwoven-12 (**d**,**e**).

To observe the surface morphology of the fibers, nonwoven fabric and M-nonwoven-12 were observed by the scanning electron microscopy. Figure 4a–c presents SEM images of the nonwoven fabric and M-nonwoven-12. As observed from both the top view (Figure 4b) and the sectional view (Figure 4c), individual nonwoven fibers are uniformly coated with a substantial amount of PEDOT:PSS. At the same time, the gaps between the fibers are also filled with PEDOT:PSS. Figure 4d,e shows the EDS images of nonwoven fabric and M-nonwoven-12. It can be observed that element S is present in the elemental analysis of both the top and sectional views. Since the nonwoven fabric consists only of three elements (C, H, and O), this indicates that PEDOT:PSS, which contains sulfur (S), has been successfully deposited onto the nonwoven fabric. The presence of the conductive polymer PEDOT enables the nonwoven fabric to exhibit conductive properties.

### 3.3. Electrical Properties of the PEDOT:PSS Modified Nonwoven Fabric

**Figure 5:**
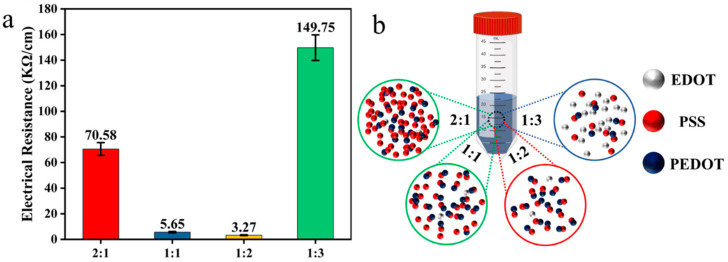
(**a**) The trend of electrical conductivity change. (**b**) Mechanism diagram of PSS doping at different ratios on PEDOT polymerization via HRP/H_2_O_2_ system.

To investigate the effect of PSS content on the electrical conductivity of PEDOT:PSS-modified nonwoven fabrics, electrical properties were systematically measured. Figure 5a shows the trend of electrical resistance. With the continuous growth of content of PSS, the electrical resistance drops first and then it increases. Compared with other samples, M-nonwoven-12 has excellent conductivity. In the reaction system, PSS serves not only as a template that facilitates the orderly and continuous arrangement of PEDOT, but also as a dispersant that ensures a uniform distribution of the monomer EDOT throughout the system (Figure 5b). However, excessive addition of PSS may counteract these beneficial effects, as PSS itself lacks electrical conductivity. Thus, in theory, the maximum electrical conductivity can be achieved when an equal or slightly excessive amount of PSS is introduced to disperse the monomer EDOT.

### 3.4. Morse Code Recognition in Piezoresistive Mode

**Figure 6:**
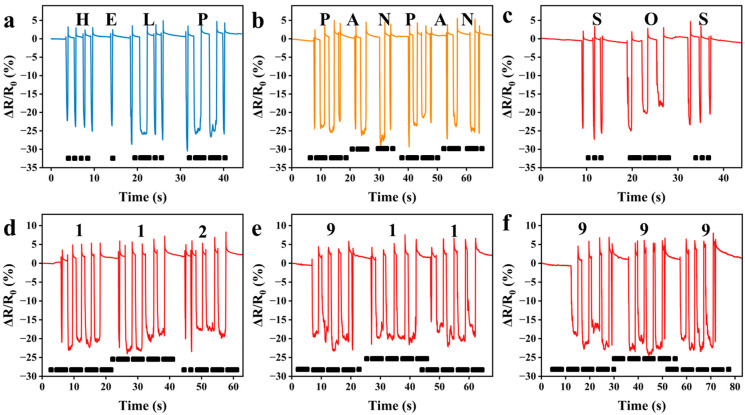
Images obtained by tapping Morse codes of letter combinations “HELP” (**a**), “PANPAN” (**b**), and “SOS” (**c**), or numeric combinations “112” (**d**), “911” (**e**), and “999” (**f**) with fingers.

Morse code, a time-honored communication protocol, encodes information through standardized sequences of dots and dashes. Notably, this technology exhibits considerable potential in the field of medical health monitoring. It functions as a silent communication interface for non-verbal individuals, enabling them to express their needs by encoding “dot/dash” sequences through fingertip presses on the fabric. This approach enables real-time, highly precise communication, illustrating the transformative potential of smart textiles within the field of assistive technology. For example, in Morse code, four consecutive dots (....) represent the letter “H”, a single dot (.) stands for “E”, the dot-dash combination (.-..) denotes “L”, and another combination (.--.) signifies “P”—these four symbols combined in sequence form the word “HELP” (Figure 6a). Furthermore, distress signals of varying urgency can be transmitted: a moderate “HELP”, a more urgent “PANPAN” (Figure 6b), and a critical “SOS” (Figure 6c). The capabilities of this system extend beyond alphabetic words. Users can also send numeric distress signals, such as emergency phone numbers in the EU for 112 (Figure 6d), North America for 911 (Figure 6e), and the UK for 999 (Figure 6f). These demonstrations clearly highlight the device’s potential as an efficient communication tool for the deaf and mute community, providing them with a reliable means to express their thoughts and needs in daily life.

### 3.5. Thermoelectric Performance

**Figure 7:**
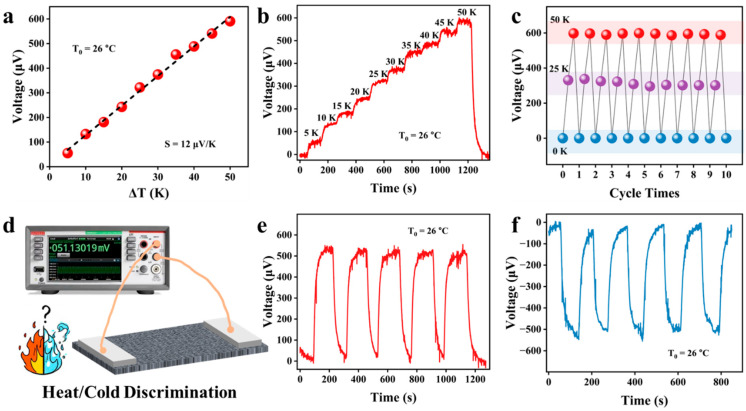
(**a**) At room temperature (T_0_ = 26 °C), the temperature difference (ΔT) between the two ends of the composite fabric shows a good linear relationship with the output thermal voltage. (**b**) The output thermal voltage of the fabric under different temperature differences which shows good differentiation. (**c**) Multiple thermal voltage response of fabrics to different kind of temperature differences. (**d**) Schematic diagram of temperature sensing test for fabrics based thermoelectric effect. By, respectively, touching one end of the fabric with a high-temperature object (**e**) and a low-temperature object (**f**), output thermal voltage signals can be generated.

Figure 7a shows that, at room temperature (T_0_ = 26 °C), there is a good linear relationship between the temperature difference (ΔT) across the composite fabric and the output thermal voltage. The slope of the curve is 12 μV/K, which corresponds to the Seebeck coefficient of the composite fabric. Therefore, temperature sensing can be realized by maintaining a constant temperature at one end of the composite fabric while varying the temperature at the other end. The study systematically investigated the output voltage response characteristics by establishing a temperature gradient ranging from 0 to 50 K between the two ends of the device. Experimental data indicate that, as the temperature difference gradually increases from 0 K to 50 K, the output voltage of the sensor exhibits a linear growth trend, eventually reaching nearly 600 μV (as shown in Figure 7b). These results clearly demonstrate the sensor’s exceptional capability to detect and respond to temperature variations across a broad temperature range.

As shown in Figure 7c, temperature differences (0 K~25 K~50 K) were applied to the sensor to record its output thermal voltage. After ten cycles, the output signal of the device remained stable and reliable. Figure 7d presents a schematic diagram of the temperature sensing performance test conducted on the device based on the thermoelectric effect. As shown in Figure 7e, when a high-temperature object approaches one side of the device, a positive thermoelectric voltage is immediately generated. Conversely, when one end of the device is brought close to a low-temperature object, a negative thermoelectric voltage is rapidly generated within the device due to the Seebeck effect (Figure 7f). The excellent reproducibility of the temperature recognition process, as demonstrated through multiple repeated experiments, effectively confirms the device’s stable and reliable temperature sensing performance.

### 3.6. Morse Code Recognition in Thermoelectric Mode

**Figure 8:**
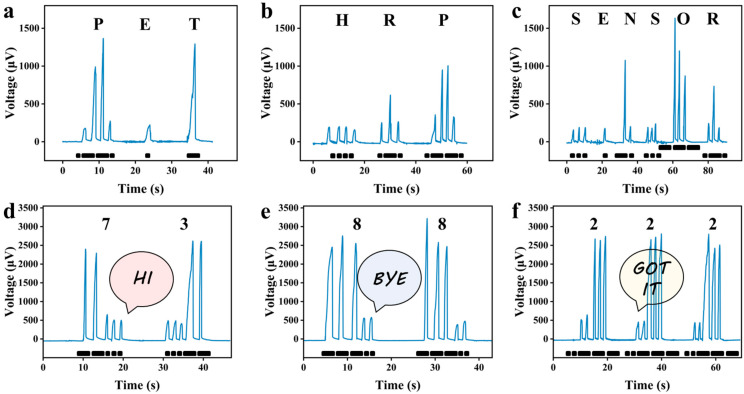
Images obtained by Morse codes of letter combinations “PET” (**a**), “HRP” (**b**), and “SENSOR” (**c**), or numeric combinations “73” (**d**), “88” (**e**), and “222” (**f**), composed of different output thermal voltages generated by touching with 1–2 fingers.

Based on the Seebeck effect, when two different conductive materials form a closed loop with a temperature difference at both ends, a thermoelectromotive force proportional to the temperature difference will be generated in the loop. Thermoelectric temperature sensing accurately measures the thermoelectromotive force to determine both the actual temperature difference between the sensor and its surroundings, and the true temperature of the target object. Thus, leveraging its excellent temperature detection and recognition capabilities, a self-powered telegraph was successfully developed. As shown in Figure 8a, “dot-dash-dash-dot” represents the letter “P”, “dot” represents “E”, and “dash” represents “T”. The combination “PET” is exactly the abbreviation of the base material used in this study, Polyethylene Terephthalate (PET). Similarly, professional terms such as “HRP” (horseradish peroxidase) and “Sensor” can also be transmitted (Figure 8b,c). In digital communication scenarios, the combinations “73” (Figure 8d) and “88” (Figure 8e) signify friendly greetings and farewells, respectively, while “222” is commonly used by radio enthusiasts to indicate “message received” (Figure 8f).

### 3.7. Morse Code Recognition in Collaborative Mode

**Figure 9:**
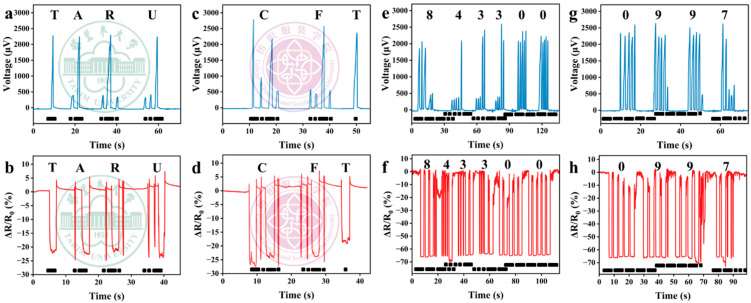
Response to changes in voltage and relative current when transmitting and recognizing Morse codes of letter combinations “TARU” (**a**–**b**) and “CFT” (**c**–**d**), or numeric combinations “843300” (**e**–**f**) and “0997” (**g**–**h**) in collaborative mode.

Based on the aforementioned research findings, we have designed and developed an innovative dual-mode Morse code transceiver system. Through a sophisticated synchronous monitoring mechanism, the system can capture and analyze in real time the dynamic characteristics of voltage signals and the rate of resistance change, thereby enabling efficient encoding and precise decoding of letters and numbers. This provides users with a flexible and reliable channel for emotional communication and information transmission. The system demonstrates remarkable versatility in practical application scenarios. As shown in Figure 9a-d, through specific signal combinations, the system can accurately recognize the abbreviation codes for “Tarim University” and “College of Fashion and Textiles,” clearly illustrating its information-expression and institutional-identification capabilities in campus environments. In the field of geographic information encoding, the numeric sequence “843300” transmitted in Figure 9e,f corresponds to the postal code of the location of Tarim University, whereas “0997” displayed in Figure 9g,h accurately represents the telephone area code of Alar City, where the university is situated. Through the deep mapping between digital signals and geographic identifiers, the system fully highlights its innovative application value in practical scenarios such as address encoding and regional information transmission. This achievement not only extends the application scope of Morse code within modern communication technologies, but also offers novel technical insights and practical cases for fields such as wearable devices and barrier-free communication, by virtue of its dual-mode signal monitoring mechanism design.

## 4. Conclusions

In this study, a PEDOT conductive finishing solution exhibiting excellent electrical conductivity was successfully synthesized through a green and straightforward approach, and the influence of PSS on the conductivity of the solution was systematically investigated. As an anionic long-chain polymer, PSS itself is electrically non-conductive and primarily functions as a dispersant and templating agent. The experimental results indicate that both insufficient and excessive amounts of PSS can significantly increase the resistance of the conductive finishing solution, thereby markedly reducing its electrical conductivity. When the molar ratios of PEDOT to PSS are 1:1 and 1:2, respectively, the finishing solution demonstrates extremely low resistance values. Additionally, this study verified another hypothesis: through an impregnation process, PEDOT:PSS was uniformly loaded onto the surface of nonwoven fabrics. The prepared flexible conductive nonwoven fabric can not only effectively recognize Morse code in either single-mode (piezoresistive or thermoelectric) operation or through collaborative dual-mode sensing, but also accurately monitor environmental temperature changes due to its sensitive temperature-responsive characteristics. This research achievement provides a new strategy for the green preparation of conductive finishing solutions. The prepared conductive finishing solution and its derivative materials, with their multifunctional properties, exhibit enormous application potential in cutting-edge fields such as intelligent sensing, human-computer interaction, and flexible electronics.

## Figures and Tables

**Table 1:** The addition proportion of PSS and EDOT and the products’ names.

Samples	EDOT (mmol/L)	PSS (mmol/L)
PEDOT:PSS-21	50	25
PEDOT:PSS-11	50	50
PEDOT:PSS-12	50	100
PEDOT:PSS-13	50	150

## Data Availability

All the data generated or used during the study appear in the submitted article.
